# Spatial Network Connectivity and Spatial Reasoning Ability in Children with Nonverbal Learning Disability

**DOI:** 10.1038/s41598-019-56003-y

**Published:** 2020-01-17

**Authors:** Sarah M. Banker, Bruce Ramphal, David Pagliaccio, Lauren Thomas, Elizabeth Rosen, Anika N. Sigel, Thomas Zeffiro, Rachel Marsh, Amy E. Margolis

**Affiliations:** 1 The Division of Child and Adolescent Psychiatry in the Department of Psychiatry, the New York State Psychiatric Institute and the College of Physicians & Surgeons, Columbia University, 1051 Riverside Drive, New York, NY 10032 USA; 2 University of Maryland School of Medicine, Baltimore, MD 21201 USA; 3 Neurometrika, Potomac, MD 20854 USA

**Keywords:** Spatial memory, Social behaviour, Human behaviour

## Abstract

Nonverbal Learning Disability (NVLD) is characterized by deficits in visual-spatial, but not verbal, reasoning. Nevertheless, the functioning of the neural circuits supporting spatial processing have yet to be assessed in children with NVLD. We compared the resting state functional connectivity of a spatial brain network among children with NVLD, children with reading disorder (RD), and typically developing (TD) children. Seventy-five participants (7–15 years old) were included in the study (20 TD, 24 NVLD, and 31 RD). Group differences in global efficiency and functional connectivity among 12 regions comprising a previously defined spatial network were evaluated. Associations with behavior were explored. Global efficiency of the spatial network associated positively with spatial ability and inversely with socioemotional problems. Within the spatial network, associations between left posterior cingulate (PCC) and right retrosplenial cortical activity were reduced in children with NVLD relative to those without spatial deficits (RD and TD). Connectivity between left PCC and right posterior cerebellum (Crus I and II) was reduced in both groups of children with learning disabilities (NVLD and RD) relative to TD children. Functional connectivity of the spatial network was atypically associated with cognitive and socioemotional performance in children with NVLD. Identifying a neurobiological substrate for NVLD provides evidence that it is a discrete clinical entity and suggests targets for treatment.

## Introduction

Nonverbal Learning Disability (NVLD) is a neurodevelopmental disorder characterized by deficits in spatial, but not verbal, reasoning. Children with NVLD frequently have accompanying impairments in socioemotional functioning, mathematical skills, executive function, and fine motor control^1–3^ that may derive from their core deficit in spatial processing. Understudied is if NVLD is discrete from other neurodevelopmental disorders. For example, the social deficits associated with the disorder are often thought to overlap with Autism Spectrum Disorders (ASD). However, prior findings point to differing social deficits in the two disorders^1,2^ that are subserved by distinct circuit alterations^4^. A deeper understanding of the neural correlates of NVLD could provide evidence for recognizing NVLD as a discrete clinical entity.

Spatial reasoning is a complex cognitive skill that relies on perception, memory, attention, and object recognition^5^. The spatial deficit in NVLD encompasses problems in visuospatial awareness (e.g., awareness of own body in space), visuospatial construction (e.g., copying visually presented materials), visuospatial memory (e.g., remembering patterns and designs), spatial estimation (e.g., judging distance), three-dimensional thinking (e.g., imagining how things will look when rotated), interpreting information presented pictorially (e.g., reading maps) or visuospatial attention (e.g., visual scanning)^6–10^. Spatial function is also known to associate with social function^11,12^, suggesting that the social impairment observed in NVLD may derive from core deficits in spatial dysfunction. For example, children with NVLD might have difficulty in social situations due to their inability to comprehend nonverbal communication or cues, or to judge interpersonal space^13,14^. Despite the documented spatial deficits in NVLD, the functioning of the neural circuits that support spatial processing have yet to be assessed in children with NVLD. Prior findings using task- and resting state functional connectional connectivity point to the existence of a spatial orientation decision network^15^. In the current study, we examined resting state connectivity of this spatial network in children with NVLD. To avoid behavioral confounds associated with differential group performance in spatial tasks, we elected to study the spatial network using resting state functional connectivity rather than task-fMRI.

Herein, we compared resting state functional connectivity in children with NVLD to typically developing (TD) children and a clinical control group, children with reading disorder (RD). Children with RD have strengths in spatial reasoning^16–19^ despite other learning deficits^20^. Contrasting these groups thus allowed us to isolate functional abnormalities specific to NVLD from those associated with learning disabilities more generally. We constructed a spatial network using previously identified regions of interest (ROIs) that were activated during a spatial orientation decision task in healthy adults; resting state functional connectivity between these ROIs predicted spatial task performance in these same individuals^15^. Meta-analyses of spatial task fMRI studies have identified similar regions to those in the selected network^21,22^, providing strong evidence for their involvement in spatial reasoning. We first attempted to demonstrate the existence of the spatial network in children, extending prior work in adults^15^. We then assessed spatial network global efficiency, a graph theoretical measure of network efficiency. We hypothesized that spatial ability (as measured by Performance Intelligence Quotient [PIQ], composed of Block Design and Matrix Reasoning subtests) would be positively associated with resting state functional connectivity of the spatial network. Second, we evaluated group differences and hypothesized that children with NVLD would show altered global efficiency and region-to-region connectivity within the spatial network relative to RD and TD children, consistent with the spatial deficits that define NVLD. Last, we explored associations of socioemotional function (Child Behavior Checklist [CBCL], Total Problems and Total Competence subtests) with spatial ability (PIQ), and associations of both of these processes with spatial network connectivity in children with NVLD.

## Results

### Participants and behavioral test performance

All children were 7–15 years old; children with NVLD were older on average than those with RD and TD children (Table 1). NVLD and RD children had lower full-scale IQ (FSIQ) than TD children. As expected, those with NVLD had lowered spatial performance (PIQ Mean Difference = 20.74 vs. RD and 31.96 vs. TD), more parent-reported socioemotional problems (CBCL Total Problems Mean Difference = 11.99 vs. RD and 19.14 vs. TD), and lowered parent-rated competence (CBCL Total Competence Mean Difference = 13.40 vs. TD). During the resting state runs, no differences in mean head motion or number of useable images were detected between groups (Table 1).

**Table 1:** Demographic Information.

Demographics	TD (n = 20)	RD/RD-ADHD (n = 31)	NVLD (n = 24)	ANOVA F statistic
Age – months (SD)	116.70 (15.06) 86–139	120.81 (20.58) 84–155	140.83 (29.87) 87–185	7.52**
Sex – N (%) female	10 (50%)	16 (51.6%)	10 (42.7%)	0.28
FSIQ	125.75 (13.09) 88–148	111.06 (14.5) 88–134	96.38 (10.17) 78–120	28.44***
VIQ	125.00 (11.72) 95–144	110.84 (13.63) 74–141	105.25 (11.34) 82–122	14.43***
PIQ	120.25 (13.29) 84–143	109.03 (16.77) 77–141	88.29 (10.05) 70–114	30.20***
CBCL Total Problems	44.95 (8.42) 34–69	52.10 (10.96) 25–71	64.08 (8.36) 42–82	22.49***
CBCL Total Competence	53.10 (10.03) 37–70	43.38 (10.23) 28–70	39.71 (7.72) 25–55	11.23***
Usable Images	214.30 (59.22) 106–273	186.90 (68.96) 89–278	186.92 (55.44) 98–276	2.42
Mean Motion	0.22 (0.21) 0.07–0.94	0.26 (0.17) 0.06–0.74	0.40 (0.31) 0.08–1.37	1.42

Displays demographic information for typically developing (TD) children and children with Reading Disorder (RD) or with Nonverbal Learning Disability (NVLD). Means, standard deviations, and ranges are presented for all continuous variables. The ANOVA column indicates ANOVA F-statistics comparing across all three groups. The number and percent of female participants was presented in the sex row and group differences were tested using chi-squared.

FSIQ = Full Scale IQ; VIQ = verbal IQ; PIQ = performance IQ.

*p < 0.05, **p < 0.01, ***p < 0.001.

To investigate whether the socioemotional difficulties observed in NVLD might derive from spatial processing deficits, the hallmark cognitive dysfunction in NVLD, the association between spatial ability (PIQ) and overall socioemotional functioning (CBCL Total Problems and Total Competence, normed t-scores) was evaluated. Across all participants, reduced spatial ability was associated with CBCL Total Problems (b = −0.22, 95% confidence interval [CI]: −0.37, −0.07, t(68) = −2.910, p < 0.005) and Total Competence (b = 0.22, 95% confidence interval [CI]: 0.07, 0.37, t(66) = 2.90, p = 0.005) controlling for age, sex, and NVLD diagnosis.

### Spatial network in children

To demonstrate the existence of a spatial processing network in children, we examined average within network connectivity across the 12 nodes of the pre-defined spatial network. The average inter-regional connectivity of the spatial network was non-zero (mean = 0.09; 95% CI 0.083–0.106; t(74) = 16.38).

**Figure 1:**
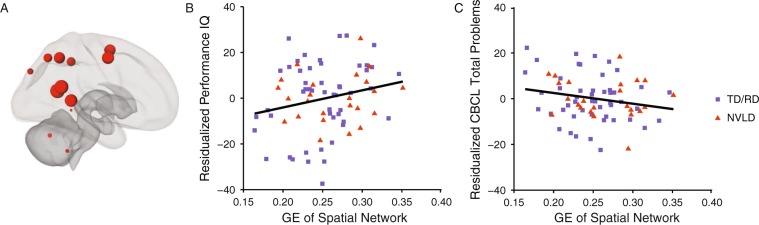
Global Efficiency of the Spatial Network. Displays (**A**) regions of interest comprising the spatial network depicted by red spheres. The relative size of the circle reflects the global efficiency (GE) of the region. Scatter plots show significant associations between residualized GE values (controlling for age, sex, mean motion, and group status) and (**B**) spatial ability (Performance Intelligence Quotient [PIQ]; b = 80.51, t(69) = 1.96, p = 0.05), and (**C**) socioemotional impairment (Child Behavior Checklist, Total Problems; b = −50.93, t(68) = −1.81, p = 0.07). Typically developing children (TD) and children with reading disorder (RD) are shown as purple squares, and children with nonverbal learning disability (NVLD) are shown as red triangles.

We then probed associations between the global efficiency of the spatial network and behavioral outcomes. GE of the spatial network was associated with PIQ (b = 80.51 [95% CI: −1.42, 162.44], t(69) = 1.96, Fig. 1) and with CBCL total problems (b = −50.93 [95% CI: −107.01, 5.14], t(68) = −1.81, Fig. 1), but not with CBCL Total Competence (b = 26.46 [95% CI: −31.18, 84.11], t(66) = 0.92, p = 0.36).

### Group differences in spatial network

**Figure 2:**
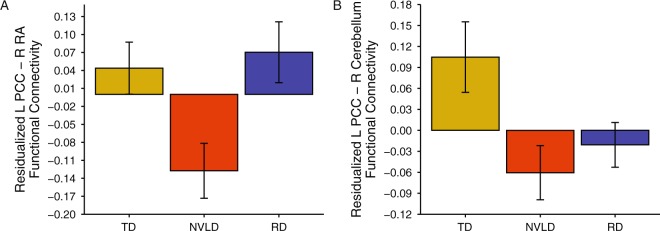
Group Differences in Spatial Network Connectivity. Displays differences in spatial network connectivity across the typically developing (TD) children, children with reading disorder (RD), and children with nonverbal learning disability (NVLD). (**A)** Shows a group difference in residualized connectivity between left posterior cingulate cortex (PCC) and the right retrolimbic area (RA). (**B)** Shows a group difference in residualized connectivity between left PCC and right cerebellum. Bars indicate mean connectivity values (Fisher r-to-Z transformed correlation values) residualized for age, sex, and mean motion; error bars indicate the standard error of the mean.

Multivariate analyses of covariance (MANCOVA), one per spatial network ROI, revealed altered spatial network connectivity with the Posterior Cingulate Cortex (PCC). No other spatial network seeds exhibited altered spatial network connectivity. Post-hoc comparisons revealed group differences between left PCC and right RA, deriving from reduced connectivity in children with NVLD relative to the other two groups (F(2, 69) = 6.82; Fig. 2), and between left PCC and right cerebellum, deriving from reduced connectivity in children with NVLD and those with RD relative to TD children (F(2, 69) = 5.15; Fig. 2). No significant group differences in spatial network GE were detected (F(2, 69) = 2.22).

### Exploratory analyses

**Figure 3:**
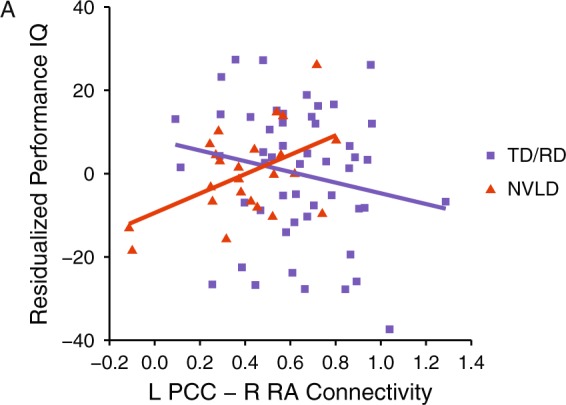
Association between PIQ and Spatial Network Connectivity. Displays the association between residualized spatial ability (Performance IQ) and left PCC - right RA connectivity (controlling for age, sex, mean motion, and group status). Typically developing (TD) children and children with reading disorder (RD) are shown as purple squares, and children with nonverbal learning disorder (NVLD) are shown as red triangles. The group by connectivity interaction predicted PIQ (b = 41.13, t(68) = 2.43, p = 0.02). Specifically, PIQ increased as connectivity increased in children with NVLD (b = 23.59, t(19) = 2.36, p = 0.03), whereas PIQ decreased as connectivity increased in TD children and children with RD (b = −12.13, t(46) = −1.37, p = 0.18). PCC = posterior cingulate cortex; PIQ = Performance IQ; RA = retrolimbic area.

To explore brain-behavior associations diagnostic groups were combined when they did not differ in connectivity. For PCC-RA connectivity, children with RD and TD children were combined, representing a group without spatial deficits, and compared to children with NVLD. The group (NVLD vs. TD + RD) by PCC-RA connectivity interaction significantly predicted PIQ (b = 41.13, 95% CI: 7.33, 74.94, t(68) = 2.43), such that PIQ increased as connectivity increased in children with NVLD (b = 23.59, 95% CI: 2.64, 44.53, t(19) = 2.36), but decreased as connectivity increased in TD children and children with RD (b = −12.13, 95% CI: −32.38, 6.12, t(46) = −1.37; Fig. 3). No statistically significant interactions or main effects were detected predicting CBCL effects. To explore brain-behavior associations of PCC-cerebellum connectivity, children with NVLD and RD were combined, representing a learning disability [LD] group, and compared to the TD children. There were no significant interactions or main effects predicting PIQ or CBCL.

## Discussion

This was the first study to examine resting state functional connectivity of a spatial network in children with NVLD, and to compare their connectivity to that in children with RD and TD children. We established a spatial network in 7–15-year-old children, extending prior work in adults, by first identifying functionally connected network regions, and second by showing that GE of the spatial network associated with PIQ across the sample. We further showed that spatial processing and spatial network GE were positively associated with socioemotional functioning, supporting the theory that deficits in spatial processing may underlie the social impairment observed in NVLD^13,14^. Finally, inter-regional connectivity was altered in children with NVLD. Specifically, cortico-cortical connectivity between left PCC and right RA was reduced in children with NVLD relative to those without spatial deficits (children with RD and TD children) and was associated differentially with spatial ability. In contrast, cortico-cerebellar connectivity between left PCC and right cerebellum (Crus I and II) was reduced in both children with NVLD and children with RD relative to TD children. These findings suggest that the spatial and social deficits in NVLD may derive from underlying alterations of a spatial processing network, providing evidence that NVLD is a discrete clinical entity.

Children with NVLD showed reduced cortico-cortical connectivity within the spatial network relative to children with RD and TD children. Associations between left PCC - right RA connectivity and spatial ability varied with NVLD diagnosis. Although connectivity between these regions increased with better spatial ability in children with NVLD, in those who did not have spatial deficits (children with RD and TD children), connectivity decreased with better ability. The retrosplenial cortex supports allocentric representation and contextual memory, aspects of spatial reasoning that may support performance on tests of spatial ability, such as PIQ^23^. Our findings may be interpreted as showing that children with RD and TD children require less cortico-cortical connectivity to achieve performance on measures of spatial ability, possibly because a level of automaticity has already been achieved.

Children with both NVLD and RD showed reduced cortico-cerebellar connectivity relative to TD children. That such altered connectivity between left PCC and right posterior cerebellum characterized both groups of children with learning disabilities points to a possible marker of learning disabilities in general. Such findings are consistent with the role of the posterior cerebellum in general learning processes, including spatial learning^24–26^. Future studies should further investigate the specific contribution of cerebellar Crus I and II functional connectivity to learning in children.

Altered connectivity in the PCC may represent a neural signature of NVLD. The PCC is a hub of the default mode network, which is known to underlie mentalizing and social processing^27,28^. As children with NVLD often have difficulties with internalizing and social problems^6,29^, such findings suggest that altered connectivity within and between the spatial and default mode networks may contribute to socioemotional problems that accompany NVLD. This interpretation is consistent with prior findings that altered connectivity between nodes of the spatial (parahippocampal gyrus) and default mode (PCC) networks were associated with social impairments in children with Autism Spectrum Disorder (ASD)^30^. Specifically, children with ASD showed increased connectivity from PCC associated with social impairment, in contrast to reduced connectivity detected in children with NVLD. These differences in patterns of functional connectivity and social impairment align with prior findings that social deficits in ASD and NVLD derive from altered patterns of connectivity within different regions of the salience network^31^.

Cortical and cerebellar regions included in the spatial network we studied are purported to support maze navigation, route learning and spatial processing in healthy individuals^32–34^ and preclinical models^24,35,36^. Task fMRI studies have shown engagement of the parahippocampal gyrus, retrosplenial cortex, and posterior parietal cortex during a virtual maze navigation task^32^ and activation of the left medial frontal gyrus and retrosplenial cortex during a route-learning task^33^. Structural MRI studies have shown that decreased cortical thickness in the precuneus, SOG, and IPL is associated with decreased spatial relative to verbal ability^34^. Evidence from rodent models also implicates a number of these regions in spatial processing, e.g. the retrosplenial cortex/posterior cingulate cortex^35,36^ and cerebellum^24^ in maze navigation. Further support for a spatial processing role involving this network derives from studies of individuals with deficits in spatial reasoning. Altered regional functional activity in this spatial network has been documented in individuals with Turner syndrome^37^, 22q11 deletion^38^, and neurofibromatosis^39^, conditions characterized by spatial impairment. Consistent with our finding of altered PCC connectivity in those with NVLD individuals with 22q11 deletion show reduced PCC activity during spatial working memory tasks^40^. In contrast to our finding of reduced connectivity between PCC-posterior cerebellum in individuals with LDs, individuals with neurofibromatosis show increased connectivity between PCC and cerebellar regions^39^. These contrasting findings may point to differences in pathophysiology of the two disorders.

Our study has several limitations. First, the relatively small sample size limits the generalizability of findings. Second, exploratory associations between behavioral outcome measures and functional connectivity values were not corrected for multiple comparisons and therefore require replication. Third, we did not have direct measures of social processing across all of children; future studies should include a broader range of spatial and social tasks to further understand the processing deficits in children with NVLD. In addition, future studies with larger samples may be successful in disentangling functional alterations in spatial versus reading circuits in children with LDs, thereby dissociating such alterations based on behavioral phenotypes.

In sum, the present study investigated, for the first time, the neural underpinnings of the spatial deficit that characterizes NVLD. We provided evidence that the spatial network observed in adults is also present in children, and that efficiency of this network associates with spatial ability and socioemotional functioning. In addition, we demonstrated that children with NVLD have altered functional connectivity within this network that associates with the spatial impairments that characterize the disorder, suggesting that this pattern of aberrant connectivity may represent a neural signature of NVLD. These findings may guide novel opportunities for treatment, e.g. behavioral treatments could target remediation of spatial deficits and assess subsequent improvement in spatial and socioemotional functioning. Additionally, pharmacological targeting of the spatial network may likewise improve function in these areas.

## Methods

### Participants

One hundred and two children (7–15 years old) enrolled in the current study and were screened for inclusion/exclusion at the New York State Psychiatric Institute, including three groups of children (50 children with NVLD, 63 children with reading disorder, and 22 typically developing children) that were recruited through announcements posted at local schools and clinics, on social media, and in the newsletter of The NVLD Project, a non-profit organization aimed at developing resources for families of children with NVLD. All children were monolingual English speakers. The Institutional Review Board at New York State Psychiatric Institute approved the study; children and their parents and/or legal guardians provided written informed assent and consent, respectively. All research was performed in accordance with the relevant guidelines and regulations.

Of the 50 children evaluated for NVLD (see below), 15 did not meet diagnostic criteria, and five others did not successfully complete an MRI scan (refused to scan, aborted during scan, and/or fell asleep), leaving 30 children with NVLD. Of the 63 children evaluated for RD (see below), eight did not meet diagnostic criteria and 21 did not successfully complete an MRI scan, leaving 34 children with RD. Of the 22 TD children recruited, none met exclusionary criteria and one did not successfully complete an MRI scan, leaving 21 TD children. Of the children who met criteria and completed an MRI scan, 6 children with NVLD, 3 children with RD, and 1 TD children were then excluded from imaging analyses due to head motion (see below). A total of 24 children with NVLD, 31 children with RD, and 20 TD children were included in the final analyses (Table 1).

### Diagnostic criteria

A diagnosis of NVLD was established in accord with prior research criteria^31,41,42^ (Table 2). Children were included in the NVLD group if they had perceptual deficits, intact reading abilities, and deficits in two of the following domains: fine motor, math calculation, visual executive functioning, or social skills.

**Table 2:** Criteria for NVLD Diagnosis.

Criterion	Assessment Measure
***Child must have:***	
Perceptual deficit *OR* a discrepancy between VIQ and PIQ (>15 points)	WISC or WASI: Block Design or Matrix Reasoning ≤ 16^th^%ile
Intact single word reading abilities	WJ-III Letter Word Identification > 16^th^%ile
Absence of autistic traits	ADI-R Interests and Behaviors Module ≤ 4
***Child must also have 2 of the following:***	
Fine motor difficulties	Perdue Pegboard ≤16^th^%ile
Math calculation difficulties	WJ-III Calculation ≤16^th^%ile
Visual executive functioning difficulties	Rey Osterrieth Complex Figure Test Copy ≤16^th^%ile
Social difficulties	Vineland-II Socialization domain ≤16^th^%ile or CBCL Social Problems ≥95^th^%ile

Displays the criteria for NVLD diagnosis. ADI-R = Autism Diagnostic Interview – Revised; CBCL = Child Behavior Checklist; NVLD = Nonverbal Learning Disability; WJ = Woodcock Johnson; WASI = Wechsler Abbreviated Scale of Intelligence; WISC = Wechsler Intelligence Scale for Children.

A diagnosis of RD was established by two independent licensed psychologists following the procedure outlined in Davis, *et al*.^43^. Children were included if an RD diagnosis was indicated by clinical history and by poor performance (at or below 25th percentile) in at least three domains: word-reading accuracy, pseudoword reading, encoding, rapid naming, or silent or oral reading comprehension. Lifetime diagnosis of neurological or neurodevelopmental disorders (other than Specific Learning Disorder or ADHD) were exclusionary, as determined by clinical interview and administration of the Kiddie Schedule for Affective Disorders and Schizophrenia (KSADS)^44^.

Typically developing children had no current or lifetime diagnoses as determined by the KSADS^44^. Children in all three groups were excluded if they had an Full-Scale Intelligence Quotient [FSIQ] < 80 based on the Wechsler Abbreviated Scale of Intelligence (WASI)^45^, any history of major medical conditions, or MRI contraindication.

### Neuropsychological and psychosocial outcome measures

A neuropsychological test battery was administered to all participants by a certified school psychologist (Ed.M.) who had formal Autism Diagnosis Interview-Revised (ADI-R)^46^ and KSADS clinical training. Measures were selected to identify clinical diagnoses. Parents of all participants completed the Child Behavior Checklist (CBCL), a measure of behavioral impairment^47^. All children completed: WASI, full scale, verbal, and performance intelligence quotient (FSIQ, VIQ, and PIQ, respectively) subscales; Woodcock Johnson Achievement (WJ) 3^rd^ edition, Letter-Word Identification, Word Attack, Spelling, and Reading Fluency subtests^48^; Comprehensive Test of Phonological Processing 2^nd^ edition, Rapid Letter Naming and Rapid Digit Naming subtests^49^; Gray Oral Reading Test 5^th^ edition^50^; Test of Word Reading Efficiency 2^nd^ edition, sight-word efficiency and phonemic decoding efficiency subtests^51^; and Gates-MacGinitie Reading Tests 4^th^ edition, reading comprehension subtest^52^. These measures were administered to identify reading problems. Children with NVLD additionally completed Purdue Pegboard^53^, Rey-Osterieth Complex Figure Test copy^54^, and WJ Achievement, Math Calculation subtest^55^. These measures were administered to identify NVLD.

### Neuroimaging acquisition

Functional and anatomical MRI data were acquired on a 3 T GE 750 scanner. Structural T1 images were collected with an 8-channel head coil using a 3D FSPGR sequence (flip angle = 11, TE = 2.6 ms, TR = 6.4 ms, 180 slices, 1 mm isotropic resolution). Two runs of resting state data were acquired with a 32-channel head coil using an echo planar imaging (EPI) sequence (flip angle = 77, TE = 30 ms, TR = 2000 ms, 34 slices, 3.5 mm isotropic resolution, 140 acquisition frames per run, 4 minutes and 40 seconds long). During the two resting state runs, participants were instructed to rest quietly with their eyes open without falling asleep. The examiner monitored that participants kept their eyes open and stayed awake during these scans using an in-scanner eye-tracking camera.

### Resting state functional connectivity preprocessing

Analysis was performed in the CONN toolbox v17.f (www.nitrc.org/projects/conn)^56^ for SPM 12. Preprocessing followed a previously published pipeline^31^ and included realignment, unwarping, centering, slice timing correction, outlier detection, segmentation of cerebral spinal fluid, gray, and white matter, normalization to the Montreal Neurological Institute (MNI) template, and 8 mm full-width half-maximum smoothing for functional images. Structural images were centered, segmented, and normalized to the MNI template. EPI data were band-pass filtered (0.008–0.09 Hz). Denoising was completed with anatomical component-based noise correction (aCompCor)^57^, regressing ten white matter and ten CSF components (detrended and despiked).

### Motion correction

To minimize effects of head motion, image frames exceeding 0.5 mm frame-to-frame displacement or frame-to-frame change in global signal change z > 3 were treated as outliers and in the first level models. In addition, 24 head motion parameters (motion + first-order derivatives + quadradic effects) were included in the first level models. To further adjust for potential effects of motion on functional connectivity measures, mean Euclidian head motion was included as a second level covariate. Participants with less than 81 useable frames were excluded from the analyses (N = 6 NVLD, N = 3 RD, N = 1 TD).

### Network selection and connectivity measures

To identify a candidate spatial network, we reviewed studies using either task or resting-state functional magnetic resonance (fMRI) to define circuits associated with spatial navigation and spatial reasoning. We based our network on a study that identified brain regions activated during a spatial orientation decision task. These regions included: bilateral precuneus, posterior cingulate [PCC], and middle frontal gyri [MFG], left inferior parietal [IPL] and superior occipital gyri [SOG], and right posterior cerebellum (crus I/II), parahippocampal gyrus, and retrosplenial cortex [retrolimbic area; RA]). Subsequently functional connectivity of this network during resting state was shown to associate with performance on the spatial orientation task^15^. Supporting our selection of this network, meta-analyses of spatial task fMRI studies identified regions that overlapped with those in the selected network^21,22^. We additionally used Neurosynth to extract meta-analytic association maps of brain activation related to the terms “spatial” and “navigation” (thresholded at p-FDR < 0.01); 10/12 seeds in our selected spatial network overlapped these maps (Table 3; www.neurosynth.org; October 3^rd^ 2019)^58^.

**Table 3:** Spatial Network Definition and Neurosynth Validation.

	Arnold Network			Neurosynth Validation	
Seed	x	y	z	Neurosynth association with ‘navigation’?	Neurosynth association with ‘spatial’?
Left MFG	−26	−4	58	Yes	Yes
Right MFG	22	−6	50	No	Yes
Right RA	10	−44	6	Yes	No
Left IPL	−36	−44	46	No	Yes
Right PHG	32	−44	−4	Yes	No
Right Cerebellum Crus II	48	−48	−46	No	No
Right PCC	20	−54	20	Yes	No
Left Precuneus	−10	−56	50	Yes	Yes
Right Precuneus	6	−68	50	Yes	No
Left PCC	−16	−58	16	Yes	No
Right Cerebellum Crus I	34	−66	−30	Yes	No
Left SOG	−42	−86	36	No	No

Lists the regions of interest (ROI) used in resting state analyses. Each ROI is part of a previously defined spatial network (Arnold *et al*., 2014). The MNI center coordinates of each ROI are indicated in the x, y, z columns. Neurosynth-based meta-analysis (p-FDR < 0.01) of the terms “navigation” and “spatial” produced association maps of brain regions activated in relevant tasks. Overlap between Arnold ROIs and association maps is indicated in the “Neurosynth Validation” columns. MFG = middle frontal gyrus; RA = retrolimbic area; IPL = inferior parietel lobule; PHG = parahippocampal gyrus; PCC = posterior cingulate cortex; SOG = superior occipital gyrus.

The first principal component of each ROI time series was computed to determine inter-regional temporal associations. The reported functional connectivity values are Fisher r-to-Z transformed correlations between 12 ROIs defining the spatial network^15^ (Table 3). Spherical ROIs with 6 mm radius were created using Marsbar^59^ centered on the peak MNI coordinates from the prior task-based results (see Supplemental Methods). GE, a graph theoretical measure of network efficiency, was calculated as the average of the inverse value of the shortest path length from each region to each other region; thus, higher GE values indicate greater network efficiency^60,61^. To avoid bias associated with selecting only one threshold for adjacency matric calculations, we calculated the GE at three different cost thresholds (0.125, 0.150, and 0.175) and then averaged these GE values^62^. We selected this range of cost thresholds because cost >0.15 has been shown to have excellent test-retest reliability in children as young as 4 years of age^56,63^. Within network connectivity strength was assessed by examining all pairwise ROI-ROI connectivity strength values for each participant.

### Statistical analyses

#### Establishing a spatial network

To establish the existence of a spatial network in this group of children, we examined average within network connectivity across the 12 network nodes of the spatial network. To probe associations between network efficiency of the network and behavioral outcomes, the association between GE of the spatial network and spatial ability (indexed by PIQ, composed of Block Design and Matrix Reasoning tests) as well as with overall socioemotional functioning (indexed by CBCL, Total Problems and Total Competence tests) was evaluated with linear regression. Analyses covaried for factors known to associate with diagnosis and functional connectivity: age, sex, mean head motion. To identify associations above and beyond effects of diagnosis, NVLD diagnostic status (NVLD vs. other [RD or TD]) was also included as a covariate.

#### Group differences

General linear models were used to test group differences (TD, RD, and NVLD children) in GE and within spatial network connectivity strength (ROI-ROI), controlling for age, sex, and mean head motion. Multivariate analyses of covariance (MANCOVA) were used to identify spatial network ROIs with group differences in network connectivity (one for each seed), corrected for multiple comparisons using False Discovery Rate (p-FDR < 0.05). Omnibus protected, post-hoc FDR-corrected F-tests evaluated group differences in every edge associated with any significant ROI. In addition, to explore which ROI pairs differed between groups and how the groups differed from each other, we present bar graphs showing mean and standard error of residualized functional connectivity from significant ROIs across groups (Fig. 2).

#### Exploratory behavioral associations

Exploratory analyses examined associations between adjacency matrix edge strengths that differed between groups and spatial ability (PIQ) and socioemotional functioning (CBCL Total Problems and Total Competence scores). We used linear regression with group, inter-regional connectivity, and their interaction to test predictors of behavioral outcomes. In these analyses, diagnostic groups that did not differ in connectivity were combined. In this way, children with RD and TD children were combined, representing a group without spatial deficits, or children with NVLD and RD were combined, representing a learning disability [LD] group). The interaction term was dropped from models when it was not significant. All models included group, age, sex, and mean head motion covariates.

## Supplementary information

Supplementary Information

## Data Availability

Data will be made available upon request.
