# Supplementary material for: Spatial Network Connectivity and Spatial Reasoning Ability in Children with Nonverbal Learning Disability
Source: Sci Rep. 2020 Jan 17;10:561. doi: 10.1038/s41598-019-56003-y (PMC6969178; doi:10.1038/s41598-019-56003-y)
Supplement: Supplementary file 1 — Supplementary Information [file 41598_2019_56003_MOESM1_ESM.docx]

**Spatial Network Connectivity and Spatial Reasoning Ability in Children with Nonverbal Learning Disability**

Sarah M. Banker^1^, Bruce Ramphal^1^, David Pagliaccio^1^, Lauren Thomas^1^, Elizabeth Rosen^1^, Anika N. Sigel^1^, Thomas Zeffiro^2,3^, Rachel Marsh^1^, Amy E. Margolis^1^*

1.The Division of Child and Adolescent Psychiatry in the Department of Psychiatry, the New York State Psychiatric Institute and the College of Physicians & Surgeons, Columbia University. 1051 Riverside Drive, New York, NY 10032.

2. University of Maryland School of Medicine, Baltimore, MD 21201

3. Neurometrika, Potomac, MD 20854

Corresponding author: Amy E. Margolis, 1051 Riverside Drive, New York, N.Y. Unit 74. 10032. Phone: 646-774-6078. Email address: amy.margolis@nyspi.columbia.edu

**SUPPLEMENTAL MATERIALS**

**Supplemental Methods**

Defining the Spatial Network

The 6mm sphere centered on -42, -86, 36 extends slightly outside the brain; as only data within the explicit brain mask were considered, this ROI was clipped and is thus was not a perfect sphere. The 12 seeds extracted from task partial least squares (PLS) analyses and used for global efficiency analyses were obtained from the authors of Arnold, et al. ^1^

References

1 Arnold, A. E., Protzner, A. B., Bray, S., Levy, R. M. & Iaria, G. Neural network configuration and efficiency underlies individual differences in spatial orientation ability. *J Cogn Neurosci* **26**, 380-394, doi:10.1162/jocn_a_00491 (2014).
